# Report of Deaths in the City of Buffalo for the Month of May, 1864

**Published:** 1864-07

**Authors:** Sandford Eastman

**Affiliations:** Health Physician


*Report of Deaths in the City of Buffalo for the Month of May*, 1864:

Whole number of deaths from disease, 138. In addition to the above, 2 still-born were reported in the city.

SEX.—Males, 88, Females, 52.

COLOR.—White, Male, 87; White, Female, 52; Colored, Male, 1.

NATIVITIES.—United States, m 47, f 36, total, 83; German States, m 19, f 4, total, 23; Ireland, m 12, f 7, total, 19; England, m 1; Scotland, f 1; Canada, m 3, f 1, total, 4; Prussia, m 2; Portugal, f 1; Unknown, m 4, f 2, total, 6. Total, Males, 88, Females, 52—140.

PARENTAGE.—American, m 13, f 8, total, 21; German, m 37, f 19, total, 56; Irish, m 20, f 16, total, 36; English, m 5, f 1, total, 6; Scotch, m 1, f 1, total, 2; French, m 1; Prussia, m 3; Austria, f 1; Canada, m 1, f 1, total, 2; Switzerland, m 1; Portugal, f 1; Unknown, m 7, f 4, total, 11. Total, Males, 88, Females, 52—140.

CONDITION.—Married, 35; Single, 91; Widows, 4; Widowers, 1, Unknown, 9. Total, 140.

LOCALITY.—City at large, 120; Hospital of Sisters of Charity, 3; Buffalo General Hospital, 3; Catholic Foundling Asylum, 7; Erie County Alms House, 4; Small-Pox Hospital, 1; Fort Porter, 2. Total, 140.

AGES.—Under 5 years, m 32, f 23, total, 55; 5 to 10 years, m 8, f 3, total, 11; 10 to 20 years, m 5, f 5, total, 10; 20 to 30 years, m 8, f 4, total, 12; 30 to 40 years, m 7, f 3, total, 10; 40 to 50 years, m 10, f 3, total, 13; 50 to 60 years, m 6, f 3, total, 9; 60 to 70 years, m 1, f 3, total, 4; 70 to 80 years, m 6, f 2, total, 8; 80 to 90 years, f 2; 90 to 100, m 1; Still-born, m 2; Unknown, m 2, f 1, total, 3. Total, Males, 88 Females, 52—140.

BY WHOM CERTIFIED.—By regular Physicians at Public Institutions, 20; by regular Physicians in city at large, 57; by irregular Practitioners, 19; by Coroner, 12; by Undertakers, 32. Total, 140.

CAUSES OF DEATH.—Accident, m 7, f 2, total, 9; accident by drowning, m 3, f 1, total, 4; ædema glottidis, f 1; congestion of brain, m 1; bronchitis, m 2; consumption, m 9, f 7, total, 16; convulsions, m 4, f 3, total, 7; croup, m 1, f 3, total, 4; cyanosis, f 1; debility, m 1, f 3, total, 4; delirium tremens, m 2; detention, m 1; diarrhœa, m 2; disease of the heart, m 3, f 1, total, 4; disease of the liver, f 1; diphtheria, m 2, f 1, total, 3; dropsy general, m 1, f 1; dropsy abominal, m 1; erysipelas, m 2; fever, f 1; remittent fever, f 1; puerperal fever, f 1; puerperal convulsion fever, f 1; scarlet fever, m 5, f 2, total, 7; typhoid fever, m 1, f 3, total, 7; typhus fever, f 1; gangrene, m 1; gun-shot wound, m 1; hæmorrhage from umbilics, m 1: inflammation of bowels, m 1, f 2, total, 3: inflammation of brain and menings, m 2, f 1, total, 3: inflammation of liver, f 2: inflammation of lungs, m 12, f 1, total, 13: inflammation of pericardium, m 1; inflammation of stomach, f 1: intussusception, m 1: infanticide, m 1; marasmus, m 4: measles, m 1, f 3, total, 4: murder, m 1: old age, m 1, f 2, total, 3· otitis, m 1; paralysis, m 1, f 1, total, 2: rupture of heart, m 1: small-pox, m 1, f 1, total. 2: suicide, m 1: syphilis, m 1: unknown m 4, f 1, total, 5: whooping cough, f 1. Deaths from diseases, Males, 86, Females, 52—138.

The following shows the number of deaths in the city in each month of the present year, the number in 1863, and the average of each month for the five years, 1859 to 1863, inclusive:

Month of January, 1864, 128, January, 1863, 115,—5 years' average, 129. February, 1864, 117, February, 1863, 94,—5 years' average, 117. March, 1864, 143, March, 1863, 104,—5 years' average, 131. April, 1864, 144, April, 1863, 121,—5 years' average, 121. May, 1864, 140, May, 1863, 132,—5 years' average, 121.

The number of deaths in the first five months of the present year, is 106 *more* than in the corresponding period of last year, and 53 *more* than the average for five years.

SANDFORD EASTMAN, M. D., Health Physician.

